# Predicting Non Return to Work after Orthopaedic Trauma: The Wallis Occupational Rehabilitation RisK (WORRK) Model

**DOI:** 10.1371/journal.pone.0094268

**Published:** 2014-04-09

**Authors:** François Luthi, Olivier Deriaz, Philippe Vuistiner, Cyrille Burrus, Roger Hilfiker

**Affiliations:** 1 Department for Musculoskeletal Rehabilitation, Clinique romande de réadaptation suvacare, Sion, Switzerland; 2 Institut de Recherche en Réadaptation, Clinique romande de réadaptation suvacare, Sion, Switzerland; 3 Hôpital Orthopédique, Département de l’Appareil Locomoteur, Lausanne University Hospital, Lausanne, Switzerland; 4 Institute of Social and Preventive Medicine, Lausanne University Hospital, Lausanne, Switzerland; 5 School of Health Sciences, University of Applied Sciences and Arts Western Switzerland Valais, Sion, Switzerland; Ben-Gurion University of the Negev, Israel

## Abstract

**Background:**

Workers with persistent disabilities after orthopaedic trauma may need occupational rehabilitation. Despite various risk profiles for non-return-to-work (non-RTW), there is no available predictive model. Moreover, injured workers may have various origins (immigrant workers), which may either affect their return to work or their eligibility for research purposes. The aim of this study was to develop and validate a predictive model that estimates the likelihood of non-RTW after occupational rehabilitation using predictors which do not rely on the worker’s background.

**Methods:**

Prospective cohort study (3177 participants, native (51%) and immigrant workers (49%)) with two samples: a) Development sample with patients from 2004 to 2007 with Full and Reduced Models, b) External validation of the Reduced Model with patients from 2008 to March 2010. We collected patients’ data and biopsychosocial complexity with an observer rated interview (INTERMED). Non-RTW was assessed two years after discharge from the rehabilitation. Discrimination was assessed by the area under the receiver operating curve (AUC) and calibration was evaluated with a calibration plot. The model was reduced with random forests.

**Results:**

At 2 years, the non-RTW status was known for 2462 patients (77.5% of the total sample). The prevalence of non-RTW was 50%. The full model (36 items) and the reduced model (19 items) had acceptable discrimination performance (AUC 0.75, 95% CI 0.72 to 0.78 and 0.74, 95% CI 0.71 to 0.76, respectively) and good calibration. For the validation model, the discrimination performance was acceptable (AUC 0.73; 95% CI 0.70 to 0.77) and calibration was also adequate.

**Conclusions:**

Non-RTW may be predicted with a simple model constructed with variables independent of the patient’s education and language fluency. This model is useful for all kinds of trauma in order to adjust for case mix and it is applicable to vulnerable populations like immigrant workers.

## Introduction

Injuries are a major public health problem that incurs huge costs [1]–[5]. Among injuries, non-fatal orthopaedic trauma is a leading cause of persistent pain, poor quality of life, long lasting sick-leave and disabilities [2], [6], [7]. As in chronic low back pain [8], only a minority of trauma patients have poor outcomes [3], [9]. As there is evidence that work has a positive impact on health, helping people returning to work is a focal point for public health [9]. Consequently, screening patients at risk of unsuccessful return to work (RTW) after orthopaedic trauma is an important issue.

In 2010, Clay and coll. published a systematic review of prognostic factors for RTW after acute orthopaedic trauma [10]. Due to the lack of factors included in more than one cohort, the level of evidence of most predictors was weak. There was strong evidence only for the level of education and blue collar work and moderate evidence for self-efficacy, injury severity and receipt of compensation as prognostic factors for the duration of work disability [10]. Since this review, some prospective studies suggested additional potential prognostic factors such as age, gender, self-employment, work injury, living in a deprived area, low income, pain intensity, pain attitudes, strong belief in recovery, health status, physical functioning or the presence of symptoms of depression [9], [11]–[13]. From these studies, it appears that broad biopsychosocial knowledge is useful to predict RTW after orthopaedic trauma.

Nevertheless, prognostic research after orthopaedic trauma has received limited attention [3], [14]–[16]. All the available models for screening patients at risk of poor outcomes were built, and are only useful, for the acute phase after trauma. After the acute phase and the usual period of recovery a large proportion of patients may then be referred to vocational facilities in case of persistent disabilities [17], [18]. However, these patients do not have the same risk of unsuccessful RTW and to date there is no useful predictive model for them. Consequently, such a model will help to better identify patients with different risk profiles and allow to test the efficiencies of risk adapted interventions in randomized control trials (RCTs) [19], [20].

To date, the vocational literature is mostly focused on factors predicting RTW for patients with low back pain or various musculoskeletal disorders [21], [22]. Some other recent prospective studies also examined this issue for trauma patients [23]–[25]. All these studies underline that a biopsychosocial approach is needed. This is most often assessed by the means of self-reported questionnaires [24], [26]. Nevertheless, modelling RTW prediction based on questionnaires may suffer from selection bias: often, only a subsample of all eligible patients is used [27] because those with poor health literacy or language fluency are excluded [27], [28]. For instance exclusion of non-native workers, a growing segment of the work forces in industrialized countries, may bias a predictive model [27], [29]. It is well known that non-native workers are a vulnerable population and may be at risk of being exposed to adverse working conditions [29], [30]. Therefore, they may have more difficulties returning to work. Another reason for the higher risk of unsuccessful RTW for this group of patients may be different cultural representations and expectations, which can be a reason for drop-outs from occupational rehabilitation [31]. An elegant strategy to overcome this problem and to include all the eligible patients may be to build a predictive model from a validated generic tool of biopsychosocial complexity not relying on language fluency. This is precisely a key feature of the INTERMED tool [32], [33], a well-studied measure of biopsychosocial complexity [34]–[37]. Moreover, the INTERMED was recently able to predict poor outcomes and unsuccessful RTW after rehabilitation [25], [38].

Therefore, the purpose of this study was to develop and validate a predictive model that estimates the likelihood of unsuccessful RTW for trauma patients who need occupational rehabilitation. This model must associate easily available potential predictors, such as gender, age, education, injury severity and pain, with biopsychosocial variables not relying on language fluency, assessed by the INTERMED.

## Methods

### Study Design

The data come from a prospective, monocentric cohort study, with a collection of biopsychosocial predictors that were (a) available at admission to a rehabilitation clinic and (b) assessable independently from the patient’s language fluency. Return to work was assessed through a questionnaire sent two years after discharge from the rehabilitation clinic; in case of non-response, two reminders were sent.

### Ethics Statement

The protocol was approved by the ethical committee of the local medical association (Commission Cantonale Valaisanne d’Ethique Médicale CCVEM 04107). Patients gave an oral informed consent and the study was conducted according to the principles expressed in the “Declaration of Helsinki”. Only demographic and usual clinical data were used and anonymously analysed. In case of disagreement, patients signed a refusal letter and were excluded. This consent procedure was approved by the ethics committee.

### Setting

This study took place in the Clinique Romande de Réadaptation (CRR) at Sion (Canton of Wallis) in the French-speaking part of Switzerland. Patients, mostly blue collar workers, with orthopaedic trauma of the back, upper or lower limb and multiple trauma were included in the study between January 1^st^, 2004 to December 31^st^, 2007 for the development sample and between January 1^st^, 2008 and April 1^st^, 2010 for the temporal validation sample. Patients are referred to the clinic from all of the French-speaking counties of Switzerland, which includes urban and industrial city centres like Geneva or mountainous and more rural regions like Wallis. Switzerland is also a country with an important proportion of immigrant workers in all sectors of the economy (for details see www.bfs.admin.ch/bfs/portal/fr/index).

### Participants

All patients, hospitalized for a rehabilitation program after an orthopaedic trauma, were eligible for this study if they had no severe traumatic brain injury at time of accident (Glasgow coma Scale ≤8), had no spinal cord injury, were capable of judgment, were not under legal custody and were not older than 62 years of age at the moment of hospitalization (considered as too old to have a reasonable chance to RTW). Most of the patients were blue collar workers and were injured after traffic, work or leisure accidents. Upper limb injuries constituted 33% of all accidents, back injuries 18%, pelvic and lower limb injuries 41% and multiple trauma 8%. Patients were sent to the rehabilitation clinic when they presented persistent pain and functional limitations incompatible with RTW (median: 9 months after the accident). The aim of the therapeutic program was to control the diagnosis and to take care of patients using an interdisciplinary approach (somatic, psychological, social and occupational) in order to reduce pain and disabilities and improve chance of returning to work (usual or adapted to impairments). The average duration of stay was 5 weeks.

### Sample Size

For assessment of statistical power in studies estimating predictor effects for binary event outcomes, the number of participants in the smallest group (i.e. RTW or non-RTW) determines the effective sample size. The usual rule of thumb is “10 to 20 events needed per candidate predictor” [39]. In our study, we had 36 potential predictors. The proportion of patients not returning to work two years after discharge is about 0.5, therefore it was estimated that we would need 1400 patients, resulting in about 700 cases. This analysis was embedded in an on-going cohort study with different research questions. In 2010, there were 1505 patients with follow-up data available, therefore we decided at this time-point to develop the model. The development model had 19 variables and we therefore would need 380 cases, i.e. 760 patients with follow-up data. In 2012, 819 patients had follow-up data and it was decided to validate the model.

### Identification of Potential Predictors

In order to avoid selection bias during the development of the prognostic model, the choice of the potential prognostic predictors was made according to the following principles. Firstly, the variables should be obtainable independently from the patient’s language fluency and health literacy [27], [28]. Secondly, the variables should be clearly defined and reproducible to enhance generalizability, avoiding the use of items that leave room for different interpretations [20].

The following variables (36 items) were therefore selected according to the literature: gender [9]; age (treated as a continuous variable) [13], [40]; education (≤9 years versus >9 years) [41], [42]; employment before injury (yes versus no) [43]; qualified work (professional certification versus no certification) [25]; marital status (living in stable partnership versus alone) [44]; litigation in relation with the accident (yes versus no) [45]; native language (French versus others) [29], [30]; work related injury (yes versus no) [9]; injury severity according to the Abbreviated Injury Scale (AIS), (rank 1 to 5; 6 =  fatal injury) [9], [10], [46], trauma localization (upper limb, lower limb, spine, multiple trauma) [3] and pain [9], [11]–[13]. Quality of life, which correlates well with self-perception of disability and feeling of recovery [6], [47] was also assessed. Pain and Quality of Life were assessed with a Visual Analogue Scale (VAS, scale range 0–100) [48]–[50]. The 20 INTERMED items (see Table 1) were also all selected as potential predictors [25], [38].

**Table 1:** Summary of the domains assessed with the INTERMED.

	History	Current state	Prognoses
**Biologic**	Chronicity	Severity of symptoms	Complications and life threat
	Diagnostic dilemna	Diagnostic challenge	
**Psychologic**	Restrictions in coping	Resistance to treatment	Mental health threat
	Psychiatric dysfunctioning	Psychiatric symptoms	
**Social**	Restrictions in integration	Residential instability	Social vulnerability
	Social dysfunctioning	Restrictions of network	
**Health care**	Intensity of treatment	Organization of care	Coordination of health care
	Treatment experience	Appropriateness of referral	

(adapted from De Jonge P et al 2003 [51], a full description of domains assessed in the INTERMED is available at: http://www.intermedfoundation.org/).

The INTERMED is an observer rated and semi-structured interview which assess the patients’ biopsychosocial complexity [32], [34], [51]. It contains 20 items grouped in 4 domains (biological, psychological, social, health care system), with each one assessed over time (past, present, prognosis). Conducted by a trained nurse, the interview for the INTERMED takes about 20 minutes and has been used in our daily clinical practice since 2003. Each question is rated on a 4-point scale from 0 to 3. A total INTERMED score ranging from 0 to 60 is calculated, whereby a higher score means a higher biopsychosocial complexity. INTERMED has been compared with a variety of other validated instruments, such as the Medical Outcomes Study 36-Item Short-Form Health Survey, the Hospital Anxiety and Depression Scale, the pain VAS, and numerous others [32], [34]. It shows high inter-rater reliability and agreement [52]. Predictive validity (for example health care needs, return to work, risk of persistent disability, and need of psychosocial interventions) was analysed in dozens of studies, using many different populations and settings, from emergency room [37] to rehabilitation [25], [38], and it also exists in several languages (English, German, Dutch, French, Italian, Spanish, Japanese for instance) (for details see: http://www.intermedfoundation.org/). The INTERMED may be used as a continuous variable (from 0–60 points), but is also available with a cut-off score (≥ 21 points) [53]. For this research, each item of the INTERMED was regarded as a potential prognostic predictor. As this study started in 2004, the 5.1 version (January 2003) was used.

### Data Collection

For the present analysis, the potential prognostic predictors were assessed within 3 days after hospitalization. All of these were prospectively recorded from the INTERMED interviews at admission and from the patient’s electronic medical chart. In order to minimize selection bias, all eligible patients were included in the study. Data was assessed by a study nurse; predictors did not depend on the mother-tongue spoken and were available for all patients in the clinic as these predictors were routinely used. To reduce loss of follow-up, two reminders were sent to the patients. The rate of non-response was similar to other studies [54], [55]. To reduce the measurement bias, the INTERMED was completed following the recommendations (for details see: http://www.intermedfoundation.org/) and other potential predictors were either administrative data or VAS.

### Outcome Measure

RTW was measured by a questionnaire 2 years after discharge. RTW was defined as return to the same or accommodated job, full time or part time, over the survey period [24].

### Selection of Model Content (Model Derivation)

The model was developed with all consecutive patients staying in the clinic during the years 2004, 2005, 2006 and 2007. Candidate predictors included in the first development model are shown in Table 2.

**Table 2:** Characteristics of the development and validation study population overall and by return to work status.

	Development sample (n = 1395)			Validation Sample (n = 819)		
	All	Non-return to work (704)	Return towork (691)	All	Non returnto work (409)	Return to work (410)
Variables	N (%)	N (%)	N (%)	N (%)	N (%)	N (%)
Not returned to work at 2 years	704 (50.5)			418 (49.9)		
Women	220 (15.8)	107 (15.2)	113 (16.4)	54 (6.6)	28 (6.8)	26 (6.3)
Worked 100% before injury	1197 (85.8)	601 (85.4)	596 (86.3)	697 (85.2)	345 (84.6)	352 (85.8)
Work related injury	659 (47.2)	373 (53)	286 (41.4)	415 (50.7)	224 (54.8)	191 (46.6)
Qualified work before injury	584 (41.9)	221 (31.4)	363 (52.5)	318 (38.8)	114 (27.9)	204 (49.8)
Higher education (>9 years)	695 (49.8)	279 (39.6)	416 (60.2)	399 (48.7)	161 (39.4)	238 (58)
Living alone	462 (33.1)	219 (31.1)	243 (35.2)	257 (31.5)	100 (24.6)	157 (38.4)
Litigation	135 (9.7)	80 (11.4)	55 (8)	85 (10.4)	45 (11.1)	40 (9.8)
Local native language:	640 (45.9)	244 (34.7)	396 (57.3)	452 (55.2)	277 (67.7)	175 (42.7)
Location : Lower limb	559 (40.1)	257 (36.5)	302 (43.7)	344 (42)	171 (41.8)	173 (42.2)
Location : Back	274 (19.6)	143 (20.3)	131 (19)	128 (15.6)	62 (15.2)	66 (16.1)
Location : Upper limb	468 (33.5)	256 (36.4)	212 (30.7)	277 (33.8)	152 (37.2)	125 (30.5)
Location : Multiple Injuries	94 (6.7)	48 (6.8)	46 (6.7)	70 (8.6)	24 (5.9)	46 (11.2)
	**mean (sd)**	**mean (sd)**	**mean (sd)**	**mean (sd)**	**mean (sd)**	**mean (sd)**
Age	43.3 (10.4)	44 (9.9)	42.6 (10.9)	43 (10.7)	44 (9.9)	42.1 (11.3)
Self-perceived quality of life (0–100)	45 (27.9)	40.2 (27.4)	49.9 (27.6)	46.8 (26.7)	41.1 (26)	52.6 (26.1)
Pain (0–100)	54.5 (25.3)	58.9 (24.1)	50 (25.7)	52 (25.1)	57.8 (23.3)	46.2 (25.5)
Severity of Injury, AIS (1–6)	2 (0.8)	2 (0.8)	2 (0.8)	2 (0.9)	1.9 (0.8)	2.1 (0.9)
INTERMED :						
Chronicity (0–3)	1.7 (0.8)	1.8 (0.8)	1.6 (0.8)	2 (0.8)	2.1 (0.7)	1.9 (0.9)
Diagnostic dilemma (0–3)	1.5 (0.6)	1.6 (0.6)	1.5 (0.6)	1.6 (0.6)	1.6 (0.6)	1.6 (0.6)
Severity of symptoms (0–3)	2 (0.2)	2 (0.1)	2 (0.2)	2 (0.2)	2 (0.2)	2 (0.3)
Diagnostic challenge (0–3)	1.5 (0.7)	1.5 (0.7)	1.4 (0.7)	1.4 (0.7)	1.5 (0.7)	1.3 (0.7)
Restrictions in coping (0–3)	1.2 (0.9)	1.4 (0.9)	1 (0.9)	1.2 (0.8)	1.4 (0.8)	1.1 (0.8)
Psychiatric dysfunction (0–3)	0.7 (0.8)	0.8 (0.9)	0.6 (0.8)	1 (0.9)	1.1 (0.9)	0.9 (0.9)
Resistance to treatment (0–3)	0.5 (0.6)	0.6 (0.6)	0.4 (0.5)	0.4 (0.6)	0.6 (0.6)	0.3 (0.5)
Psychiatric symptoms (0–3)	1.3 (0.8)	1.5 (0.7)	1.1 (0.8)	1 (0.7)	1.1 (0.7)	0.8 (0.7)
Restrictions in integration (0–3)	1.9 (1)	2.2 (0.9)	1.6 (1)	2 (1)	2.3 (0.9)	1.7 (1)
Social dysfunctioning (0–3)	0.4 (0.6)	0.5 (0.7)	0.3 (0.5)	0.6 (0.7)	0.7 (0.8)	0.4 (0.7)
Residential instability (0–3)	0.1 (0.3)	0.2 (0.4)	0.1 (0.3)	0.1 (0.4)	0.1 (0.4)	0.1 (0.4)
Restrictions of network (0–3)	0.8 (0.7)	0.9 (0.7)	0.7 (0.7)	0.6 (0.7)	0.7 (0.7)	0.4 (0.6)
Intensity of treatment (0–3)	2.1 (0.8)	2.2 (0.7)	2.1 (0.8)	2.2 (0.8)	2.3 (0.8)	2.2 (0.8)
Treatment experience (0–3)	0.4 (0.7)	0.4 (0.7)	0.4 (0.7)	0.4 (0.7)	0.4 (0.7)	0.4 (0.6)
Organization of care (0–3)	1.3 (0.8)	1.5 (0.7)	1.1 (0.8)	1.2 (0.8)	1.4 (0.8)	1.1 (0.8)
Appropriateness of referral (0–3)	0.5 (0.6)	0.6 (0.6)	0.4 (0.6)	0.3 (0.6)	0.4 (0.6)	0.3 (0.5)
Complications and life-threat (0–3)	1.4 (0.5)	1.5 (0.5)	1.3 (0.5)	1.5 (0.6)	1.7 (0.5)	1.4 (0.6)
Mental health threat (0–3)	0.6 (0.6)	0.7 (0.6)	0.5 (0.6)	0.6 (0.6)	0.7 (0.6)	0.4 (0.6)
Social vulnerability (0–3)	0.9 (0.7)	1.1 (0.7)	0.8 (0.7)	1.3 (0.7)	1.4 (0.7)	1.1 (0.8)
Coordination of healthcare (0–3)	0.3 (0.5)	0.4 (0.6)	0.2 (0.4)	0.1 (0.4)	0.2 (0.4)	0.1 (0.4)

For the development sample only patients included with complete data on all variables and for the validation sample only patients with complete data on the variables from the final model are shown. AIS: Abbreviated Injury Scale.

#### Variable selection based on random forest

To select the best subset of predictive variables, we used a random forest classification model for the prediction of non-return to work, using the R package “varSelRF” [56], [57]. Random Forest is a method that determines a consensus prediction for each observation by averaging the results of many individual recursive partitioning tree models [58], [59]. A training set of size N ( =  total sample size) is drawn from the original data using bootstrap with replacement. A classification tree is computed with this training data. We repeat that a large number of times (50′000) and the final classification is the one that appears the most frequently.

When the training set is sampled, about one-third of original observations are left out. These are used to test the classification of the trees and get an error estimate [60]. We can also get information about the importance of a given predictor by comparing this classification accuracy to what we get by randomly permuting the values of this predictor. Hence a high *Mean Decrease Accuracy* indicates high importance of the predictor.

The random forest approach has been shown to provide sets of predictors with good predictive value and to be robust against overfitting, which makes them especially useful for the evaluation of a large number of possible predictors and their potential interactions as well as their association with the outcome [61]. Because standard random forest method is prone to favour continuous predictors, we used conditional random forest, as proposed by Strobl [62].

Because of the little amount of missing values we decided not to impute the missing values [63].

### Model Performance

To evaluate the model performance we presented indices for discrimination and calibration. For discrimination, we calculated the area under the receiver operating characteristic (ROC) curve, as well as sensitivity, specificity, and positive and negative predictive values. For testing the calibration we used the Hosmer-Lemeshow test [64] and plotted the observed proportions of non-return to work against the predicted probabilities for groups defined by ranges (10%) of predicted risk as well as the slopes and intercepts [65].

### Temporal External Validation

For temporal validation, we applied the model to all consecutive eligible patients included in the years 2008, 2009, and the beginning of 2010. For this validation, the coefficients and the intercept predicted in the development sample were used to predict the probabilities of not returning to work. We presented ROC-curves, calibration plots and decision curve as well as a table with sensitivity, specificity, positive and negative predictive values.

### Decision Curve Analysis

We plotted decision curves to show the net benefit of classifying patients based on our models compared to classifying all patients as not returning to work or classifying all patients as returning to work [66]. The y-axis denotes the net benefit in the units of true positives. The x-axis indicates the threshold probability at and above which one decides that the patients will not return to work.

### Construction of the Prediction Score

Firstly, to obtain the most precise estimation of the coefficients, we recalculated them using both the development and the training sample for the clinical use. This approach is often chosen because it makes full use of the data resulting in narrower confidence intervals and more stable risk scores [67]–[69]. Secondly, we used the coefficients from the logistic regression model to build a prediction score, which provides the predicted probability of not returning to work even when treated. The formula is: Probability Risk Score: = 1/[1+ exp(− scoring function)], where the scoring function consists of the sum of all products of the coefficients and the values of the predictors. The formula is implemented into an excel-sheet, so that clinicians automatically receive the probability after entering the values of the predictors of a given patient.

All analyses were done with Stata version 13.0 (College Station, Texas 77845 USA) and with R statistical software version 2.15.3 [70] with the packages *PresenceAbsence* (version 1.1.9), *extended Forest* (version 1.6) and *varSelRf* (version 0.7–3).

## Results

For the development and validation periods, from the years 2004 to 2010, a total of 3177 patients with orthopaedic trauma have been in the rehabilitation clinic. At 2 years, the non-RTW status was known for 2462 patients (77.5%).

For the development period 2004 to 2007, 2048 patients were eligible. Out of these patients, 1505 answered to the two year follow-up questionnaire (73.5%).

**Figure 1:**
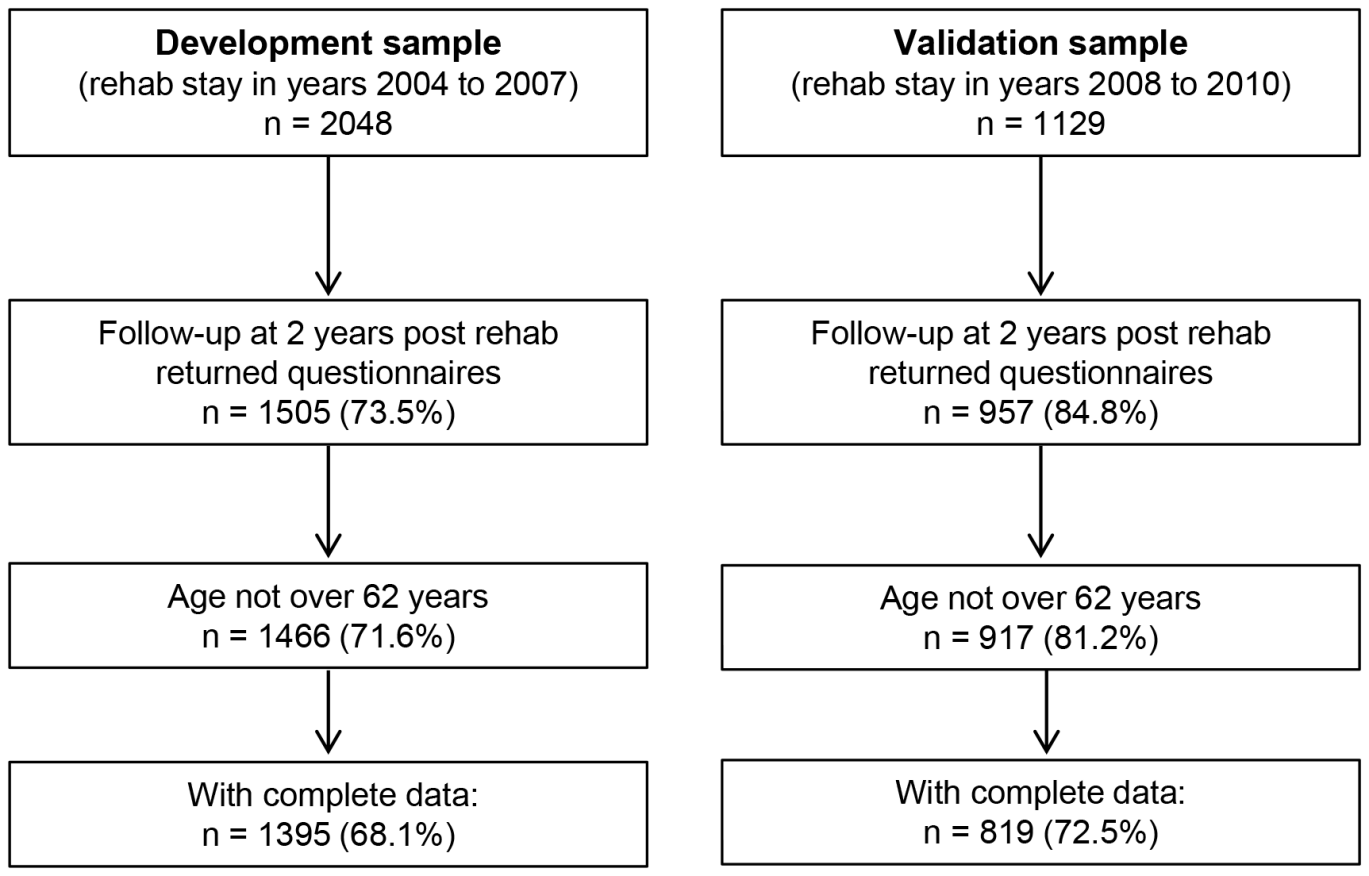
Flow chart of patients through the study.

For these analyses, 1466 patients were available. Out of these 1466 patients, 1395 had complete data for the set of predictors included in the first model. See Figure 1.

For the validation period 2008 to 2010, we had 1129 patients of which 957 returned the two years follow-up questionnaire (84.8%). We had a sample size in the validation sample of 917 of whom 819 had a complete dataset. See Figure 1.

Missing values were below 2.5% for all variables.

The baseline characteristics of the development population (n = 1395) and the validation population (n = 819) are shown in Table 2. Both samples are similar with only small, clinically non-relevant differences. For instance, 50.5% did not return to work in the development sample and 49.9% in the validation sample.

### Responders versus Non-responders

In the development sample, patients not responding to the follow-up were on average 3.4 years younger (40 versus 43.4 years, p = 0.003), more often living alone (p<0.001) and having higher values in the social (p = 0.008) and biological domains of the INTERMED (p = 0.019).

In the validation sample the only difference between responders and non-responders was age: patients not responding to the follow-up questionnaire were 2 years younger (41 versus 43 years, p = 0.008).

### Model Selection

Univariable and multivariable odds ratios for the predictors in the development sample are shown in Table 3. The random forest variable selection procedure yielded 19 variables, which were then used for the final prediction model in the development sample (shown in the last column in Table 3).

**Table 3:** Non-return to work: Odds ratios for the univariable, multivariable and the reduced model after random forest selection process.

	Univariable		Multivariable		Reduced model after conditional random forest	
Variables	Odds Ratio (95% CI)	p-value	Odds Ratio (95% CI)	p-value	Odds Ratio(95% CI)	p-value
Woman	0.89 (0.68 to 1.18)	0.431	1.03 (0.72 to 1.47)	0.873		
Age, per 10 years	1.14 (1.03 to 1.26)	0.009	1.18 (1.04 to 1.34)	0.009	1.19 (1.07 to 1.34)	0.005
Living alone	0.83 (0.66 to 1.03)	0.087	1.10 (0.83 to 1.46)	0.502		
Higher education	0.45 (0.36 to 0.55)	<0.0001	0.75 (0.55 to 1.02)	0.066	0.79 (0.59 to 1.07)	0.128
Worked 100% before injury	0.93 (0.69 to 1.25)	0.629	0.85 (0.59 to 1.22)	0.371		
Qualified work before injury	0.42 (0.34 to 0.52)	<0.0001	0.78 (0.57 to 1.06)	0.113	0.75 (0.56 to 1.01)	0.062
Work related injury	1.57 (1.27 to 1.93)	<0.0001	1.33 (1.03 to 1.74)	0.031	1.18 (0.93 to 1.5)	0.165
Litigation	1.52 (1.07 to 2.17)	<0.0001	1.25 (0.82 to 1.91)	0.29		
Local native language	0.40 (0.32 to 0.49)	<0.0001	0.64 (0.48 to 0.86)	0.003	0.67 (0.51 to 0.88)	0.004
Location : Lower Leg and Pelvis	0.75 (0.60 to 0.92)	0.006	0.65 (0.38 to 1.12)	0.122		
Location : Back	1.09 (0.84 to 1.41)	0.529	0.86 (0.47 to 1.54)	0.606		
Location : Shoulder	1.26 (1.01 to 1.56)	0.042	0.88 (0.5 to 1.56)	0.656		
Location : Multiple Injuries	1.09 (0.75 to 1.58)	0.646	1.00 (reference)			
Severity of injury, AIS	0.96 (0.84 to 1.09)	<0.0001	1.10 (0.93 to 1.30)	0.284		
Pain 0 to 100, per 10 points	1.15 (1.11 to 1.20)	<0.0001	1.05 (1.00 to 1.10)	0.068	1.04 (0.99 to 1.09)	0.114
Self-perceived quality of life 0 to 100, per 10 points	0.88 (0.85 to 0.92)	<0.0001	0.95 (0.91 to 1.00)	0.046	0.95 (0.91 to 1.00)	0.034
Chronicity	1.28 (1.13 to 1.46)	<0.0001	1.06 (0.90 to 1.25	0.506	1.04 (0.88 to 1.21)	0.661
Diagnostic dilemma	1.18 (1.00 to 1.39)	0.051	0.92 (0.74 to 1.13)	0.429		
Severity of symptoms	3.19 (1.55 to 6.56)	0.002	2.53 (1.02 to 6.24)	0.045		
Diagnostic challenge	1.41 (1.21 to 1.64)	<0.0001	1.12 (0.94 to 1.35)	0.209		
Restrictions in coping	1.53 (1.36 to 1.73)	<0.0001	1.16 (0.95 to 1.41)	0.149	1.07 (0.9 to 1.27)	0.449
Psychiatric dysfunction	1.30 (1.15 to 1.47)	<0.0001	0.82 (0.66 to 1.00)	0.054		
Resistance to treatment	2.02 (1.66 to 2.45)	<0.0001	1.10 (0.78 to 1.56)	0.58	1.07 (0.84 to 1.38)	0.572
Psychiatric symptoms	1.77 (1.53 to 2.04)	<0.0001	1.00 (0.80 to 1.24)	0.986	1.00 (0.81 to 1.23)	0.980
Restrictions in integration	1.81 (1.62 to 2.02)	<0.0001	1.41 (1.22 to 1.61)	<0.0001	1.42 (1.24 to 1.61)	<0.0001
Social dysfunctioning	1.72 (1.45 to 2.04)	<0.0001	1.17 (0.93 to 1.49)	0.184	1.10 (0.88 to 1.37)	0.426
Residential instability	1.89 (1.38 to 2.58)	<0.0001	1.34 (0.91 to 1.97)	0.135		
Restrictions of network	1.73 (1.48 to 2.03)	<0.0001	1.25 (1.01 to 1.53)	0.037	1.26 (1.03 to 1.54)	0.022
Intensity of treatment	1.19 (1.03 to 1.36)	0.015	1.14 (0.96 to 1.36)	0.148		
Treatment experience	1.04 (0.90 to 1.21)	0.59	0.94 (0.78 to 1.13)	0.496		
Organization of care	1.77 (1.54 to 2.05)	<0.0001	1.21 (1.00 to 1.46	0.05	1.28 (1.07 to 1.54)	0.008
Appropriateness of referral	1.80 (1.50 to 2.15)	<0.0001	0.93 (0.68 to 1.28)	0.671		
Complications and life-threat	2.02 (1.65 to 2.47)	<0.0001	1.23 (0.94 to 1.60)	0.133	1.29 (1.00 to 1.66)	0.052
Mental health threat	1.75 (1.48 to 2.06)	<0.0001	0.96 (0.73 to 1.26)	0.764	0.89 (0.69 to 1.13)	0.337
Social vulnerability	1.78 (1.53 to 2.06)	<0.0001	1.16 (0.95 tp 1–41)	0.15	1.19 (0.99 to 1.43)	0.071
Coordination of healthcare	2.01 (1.63 to 2.49)	<0.0001	1.30 (0.99 to 1.7)	0.059	1.23 (0.95 to 1.59)	0.117

Odds Ratios of the different models in the development sample, with corresponding 95% confidence intervals (CI).

### Model Performance

**Figure 2:**
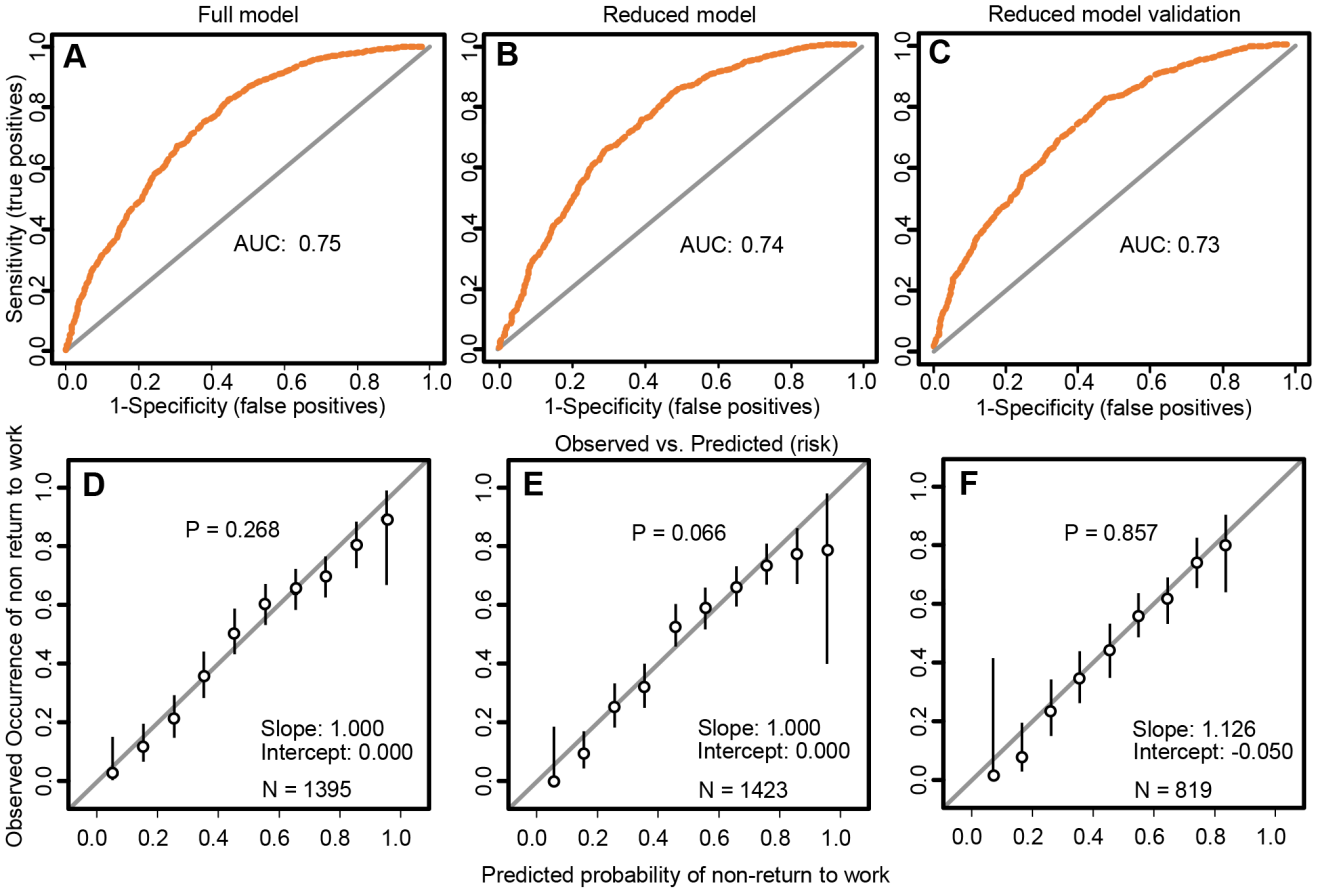
Receiver Operating Characteristic Curves (upper panel) and calibration plots (lower panel). Receiver operating characteristic curves with areas under the curves (upper panel A–C) and calibration plots (lower panel, D–F). The leftmost column is from the full model in the development sample, the middle column shows the reduced model in the development sample and the right column shows the temporal external validation of the reduced model. AUC = area under the curve. N = total number of participants with complete data for the variables in the model.

The discrimination of the reduced model after the random forest variable selection procedure was moderate (AUC 0.74; 95% CI 0.71 to 0.76) but nearly as good as the full model (AUC 0.75; 95% CI 0.72 to 0.78). In the validation sample, the discrimination of the reduced model was still sufficient with an AUC of 0.73 (95% CI 0.70 to 0.77). The calibration was good for the full model as well as the reduced model in the development and the validation sample, as indicated by the calibration plots, with p-values for the Hosmer-Lemeshow test indicating that there was no significant deviation between the observed from the predicted risk (see lower panel of Figure 2). The calibration can also be evaluated by the vertical confidence intervals in the lower panel of Figure 2: for the prediction of non-return to work, the confidence intervals of the observed probabilities (vertical black lines) covered the line of ideal calibration (diagonal grey line in the lower panel of Figure 2).

### Predictive Values

The sensitivity, specificity, and positive and negative predictive values for different cut-off points were similar in the development and the validation sample (see Table 4).

**Table 4:** Comparison predictive Values in the development and the validation sample.

Risk of not returning to work																
	Sensitivity (95% CI)				Specificity (95% CI)				Positive Predictive Value (95% CI)				Negative Predictive Value (95% CI)			
Threshold	Development		Validation		Development		Validation		Development		Validation		Development		Validation	
> = 0.1	100	(100 to 100)	100	(100 to 100)	2.6	(1.7 to 3.4)	1.71	(0.82 to 2.6)	51.3	(48.7 to 53.8)	50.4	(47.0 to 53.8)	100	(100 to 100)	100	(100 to 100)
> = 0.2	99.5	(97.8 to 99.1)	99.3	(98.7 to 99.9)	17.5	(15.5 to 19.5)	11.2	(9.7 to 14.2)	55.0	(52.4 to 57.6)	52.9	(49.5 to 56.4)	91.8	(90.4 to 93.2)	94.2	(92.6 to 95.8)
> = 0.3	93.2	(91.9 to 94.5)	94.9	(93.6 to 96.4)	33.4	(31.0 to 35.9)	26.6	(23.6 to 29.6)	58.9	(56.4 to 61.5)	56.3	(52.9 to 59.7)	82.8	(80.8 to 84.7)	83.9	(81.3 to 86.4)
> = 0.4	85.0	(83.1 to 96.9)	85.1	(82.7 to 87.5)	50.9	(48.3 to 53.2)	45.1	(41.7 to 48.5)	64.0	(62.5 to 66.4)	60.7	(57.4 to 64.1)	76.8	(74.6 to 79.0)	75.2	(72.3 to 78.2)
> = 0.5	69.6	(67.7 to 72.1)	72.4	(69.3 to 75.4)	65.2	(62.7 to 67.6)	61.2	(57.9 to 64.6)	67.2	(64.8 to 69.6)	65.1	(61.8 to 68.3)	67.8	(65.3 to 70.2)	69.0	(65.8 to 68.3)
> = 0.6	51.7	(49.1 to 54.3)	49.4	(46.0 to 52.8)	78.2	(76.1 to 80.4)	78.5	(75.7 to 81.4)	70.9	(68.5 to 73.2)	69.7	(66.5 to 72.8)	61.3	(58.7 to 63.8)	60.9	(57.5 to 64.2)
> = 0.7	30.4	(28.0 to 32.8)	26.4	(23.4 to 29.4)	89.5	(87.9 to 91.1)	92.4	(90.6 to 94.3)	74.7	(72.5 to 77.0)	77.7	(74.9 to 80.6)	55.7	(53.1 to 58.2)	55.7	(52.3 to 59.1)
> = 0.8	11.4	(9.7 to 13.0)	7.6	(5.8 to 9.4)	96.4	(95.5 to 97.4)	98.3	(97.4 to 99.2)	76.6	(74.4 to 78.8)	81.6	(78.9 to 84.2)	51.5	(48.9 to 54.1)	51.6	(48.2 to 53.0)
> = 0.9	1.0	(0.5 to 1.5)	–		99.7	(99.4 to 100)	–		77.8	(75.6 to 79.9)	–		49.6	(46.9 to 52.1)	–	

Compares diagnostic properties in the development sample with the validation sample. Threshold = Chosen cut-off for the dichotomizing in test negatives (i.e. return to work, below thresholds; non return to work, equal or above threshold).

In these samples, all patients received the traditional healthcare (usual occupational rehabilitation) which corresponds to using a cut-off of 1, meaning that everybody is considered as potentially returning to work. Using the predictive model would allow some patients to be classified as non-RTW and those would receive an adapted occupational treatment.

**Figure 3:**
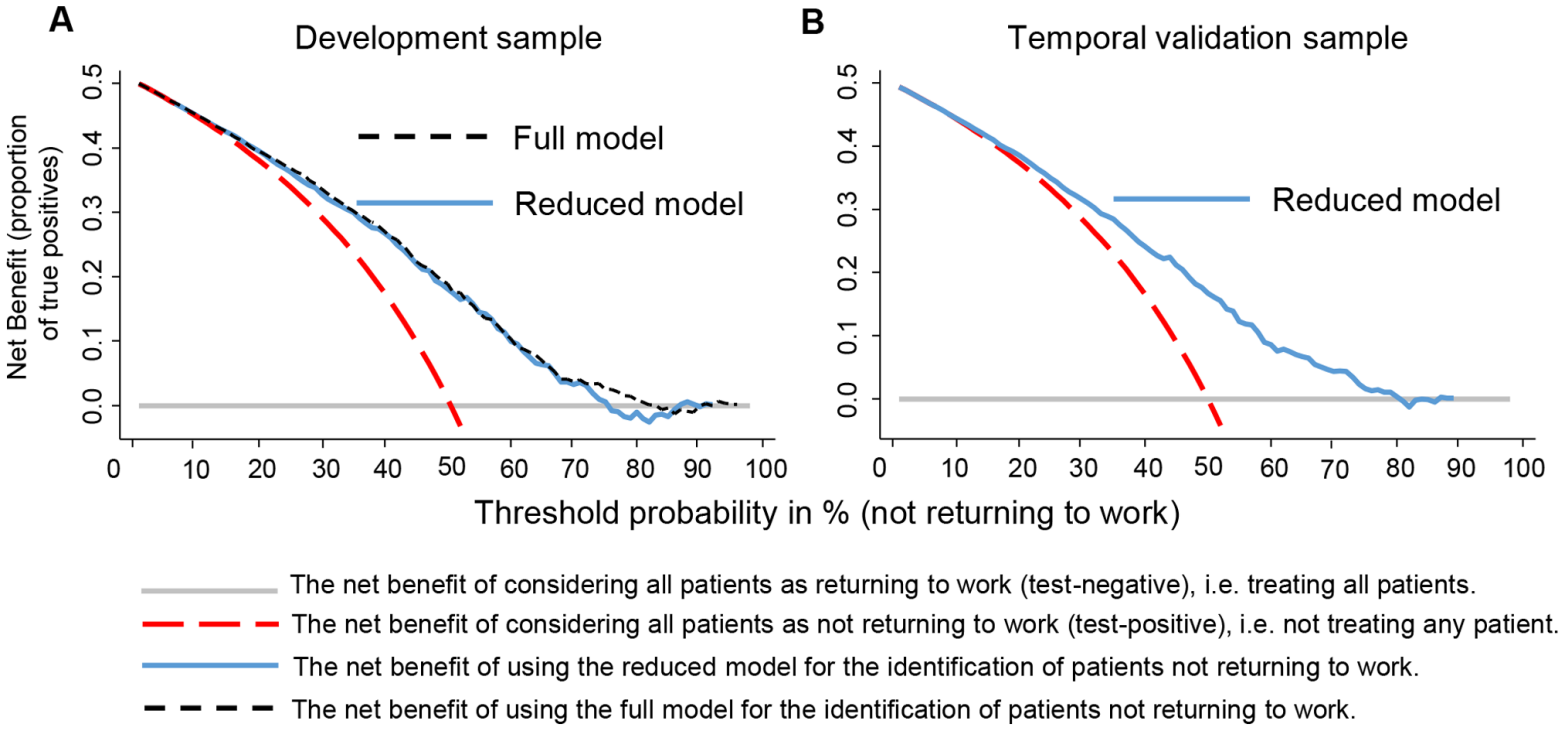
Decision curve analysis. Decision curve analysis of the Full Model (dashed line black line) and Reduced Model (blue solid line) in the development sample (Panel A) and the Reduced Model in the temporal validation sample (Panel B). The y-Axis represents the net benefit, which is the probability of true positives minus the probability of false-positives weighted for the threshold probability. With threshold probability (or risk thresholds) we mean the threshold above which a patient is declared at risk to not return to work at two years. The dashed red curve shows net benefit of considering all patients as positive (i.e. classified as being not returning to work). The benefit of considering all patients as returning to work was set as reference (solid grey horizontal line). In the left Panel (A) we see that the net benefits for both models are quite similar. The Full Modell would show advantages if a threshold would be set between 15% to 82%. The right Panel (B) shows that that the net benefit in the temporal validation sample is only little lower than in the development sample. Clear benefits are seen from risks thresholds from about 20 to 75%. The net benefit is calculated as (proportion of true positives) – (proportion of false positives)*pt/(1−pt), where pt is the threshold probability.

The net benefit for patients classified as non-RTW [66] was quite similar in the validation sample compared to the development sample. Net benefit was present for threshold probabilities of around 20% to 75% (see Figure 3). In Figure 3, the net benefit at a threshold probability of 50% is 0.16. This corresponds to the difference between the proportion of true-positives (those correctly classified as non-RTW) and the proportion of false-positives (those classified as non-RTW that actually would RTW).

In Table 5 we illustrate with the threshold of 0.5 (our choice of preference) the proportion of patients correctly or wrongly classified. Compared to the current situation (i.e. threshold of 1), by using a threshold of 0.5, in a sample of 100 patients, we correctly withheld usual occupational rehabilitation from 36 patients (i.e. true positives). This comes at a cost: we falsely withheld usual occupational rehabilitation from 19 patients (i.e. false positives). This means that a more comprehensive assessment is needed using a second step. With this comprehensive assessment, most of the false positives will be re-allocated to the usual occupational rehabilitation. In other words, for clinical use, all candidates to occupational rehabilitation should be screened with the predictive model at entry, an inexpensive and fast procedure. Then, candidates who would be above the designated cut-off point (i.e. putative true positives) should have a comprehensive assessment for a few days to recover the false positives. These patients would be reallocated to the usual occupational rehabilitation while others (true positives) would benefit from an adapted occupational approach.

**Table 5:** Proportions of true-positives (TP), false-positives (FP), true-negatives (TN) and false-negatives (FN) given by the Reduced Model in the temporal validation sample, according to threshold of 0.5 (sample with 100 patients).

	True work status at 2 years after rehabilitation		
	Non-RTW	RTW	
≥0.5 risk of non-RTW	36 TP	19 FP	55
<0.5 risk of non-RTW	14 FN	31 TN	45
	50	50	100

### Scoring of the Prediction Model

For clinical use, the scoring sheet and the prediction formula is available as supporting information: see Reduced Model S1.

## Discussion

Based on a prospective cohort of over 2000 patients, we developed and validated a simple predictive model (19 items) to estimate the probability of non-return to work after orthopaedic trauma. This model, which showed acceptable discriminative ability to assess the likelihood of non-return to work and good calibration (see Figure 2), can be applied to all patients requiring occupational rehabilitation independent of their language fluency and literacy. Consequently, unlike in most studies using questionnaires, this strategy will reduce selection bias observed in earlier studies [27] i.e. allow the assessment of *all* eligible patients including vulnerable patients with different languages and education backgrounds, like for instance immigrant workers.

This study has several strengths. To date, this is the first model available for patients suffering of persistent impairments and disabilities after orthopaedic trauma. The previous models were all reserved for the acute phase after orthopaedic trauma [3], [14]–[16]. In the acute and sub-acute phase, most of the patients will recover and those remaining with persistent disabilities will not have a similar risk profile of non-returning to work [23]–[25]. Consequently, our predictive model may improve the decision-making process if occupational rehabilitation is needed. Further strengths of our study are the large sample size, the external temporal validation and the appropriate variable selection based on random forest. For instance, random forest has clear advantages over stepwise selection methods [71]. In addition our model is constructed on the biopsychosocial framework, which adheres to the current recommendations [40], [45]. Furthermore, language fluency was not a barrier for the participation in this study.

Nevertheless, our study has also some limitations. Firstly, the potential predictors were selected ten years ago. Hence, our model may miss newer “candidates”- predictors, for instance patient’s subjective appraisal of injury severity, self-perceived disability, pain beliefs, and recovery and job expectations. However, the review of the current literature shows that the chosen predictors in the present study cover most of those cited in the recent literature [9], [11]–[13]. For instance, “living in deprived areas”, a predictor found important in the study of Kendrick [9] is close to the “social vulnerability” concept of the INTERMED. Another limitation to keep in mind is the fact that we only did a temporal validation and not a validation in a different setting in regard of the health system, the culture and the case-mix [63]. However, this disadvantage may be reduced by the fact that our patients came from many different areas of Switzerland, with various cultural backgrounds. Moreover, 50% of our sample consists of immigrant workers. Our definition of RTW and the time point of its assessment may also be questionable: firstly, we used a subjective method (questionnaire). Yet there is no clear consensus on the best way to assess RTW [72]. The risk exists to over or underestimate the RTW rate whichever method you use [73]. Nevertheless, self-report indicators are recommended to capture a fuller extent of workers’ experience [74]. In further studies, it would be necessary to define when and how long people had RTW. Secondly, the 2 years follow-up may be too long or even too short to evaluate a successful RTW. Too long because within a time frame of two years much can happen independently of the patient’s state at prediction. Too short because in this group of patients vocational reintegration and the insurance process may take longer. For instance, data of the Swiss Injury insurances suggest that it takes up to four years until the closure of the case (for details, see www.unfallstatistik.ch). In other words, further studies with different time frames to estimate RTW are also needed. Differences between non-responders may also bias the prevalence of RTW (lack of outcome data in 22.5% of our sample). It is hard to interpret these findings because differences may be influential in both directions (over or underestimation of the non-RTW rate). Nevertheless, this prevalence was quite close in both samples despite different non-responders characteristics. Our model has only moderate discriminative ability (AUC). However, predictive models have generally lower performance (AUC between 0.6 to 0.85) compared to diagnostic or explicative models (AUC >0.8) [19], [63]. Finally, our predictive model was developed and validated in a highly selected population which limits its generalizability. Further studies are needed with different time off work and access to occupational rehabilitation facilities.

Comparison with other predictive tools is limited. To our knowledge, there is no predictive model for a population with similar characteristics than ours. Predictive models during the acute phase after orthopaedic trauma are prominently based on injury severity [3], [14]–[16]. However, the importance of psychosocial factors to predict RTW increases when we move away from the accident [9], [13], [40]. The subjective perception of injury severity may also become more significant [24] than objective severity as measured by clinical tools. This is confirmed by the present study in which the severity of the accident was not included in the final model. When we compare our model with prediction models used in patients with neck or low back pain, a research domain very close to ours, we observe that their theoretical constructs follow the same biopsychosocial framework [75], [76]. We notice, however, that in low back pain the most helpful predictors of persistent disability are often issued from self-reported questionnaires [77]. In a multicultural context, this approach requires the translation and cultural adaptation of several questionnaires, which is a costly and time consuming process [78]. For this reason, we used another strategy and our predictive model shows comparable discriminative abilities than others for neck and low back pain [76], [79], [80]. The INTERMED, the essential source of our predictive model (12 items in the final model), is already available in several language (see Method section) and this is a worthwhile advantage. Moreover the remaining 7 items are all easy to translate into any languages.

Our study has some implications for practice and research. First, our model provides a short patient’s bedside tool useful to estimate the likelihood of non-return to work. Considering that a complete INTERMED interview (20 items) takes no more than 20 minutes [37], we assume that our model can be filled out in a similar time or even less. Only 12 INTERMED items have been retained in our model. The other 7 items are 5 basic medical data, easily accessible from the patient’s chart and 2 VAS evaluated by the patient. Clear instructions on how investigators should answer to the different items exist and the predictive formula may be easily programmed on electronic devices (see supporting information, Reduced Model S1). Currently, it has become customary in industrialized countries to address patients with persistent disabilities to an interdisciplinary occupational rehabilitation program [17]. Nevertheless, in our setting, this approach is unsuccessful for 50% of the patients (patients do not return to work despite vocational rehabilitation). Our model may allow a reduction of the number of unsuccessful usual traditional rehabilitations. The benefit may be to save money, but most importantly to try alternative approaches for these patients. In this way, our model may also allow to define groups of patients with similar risks of non-RTW profiles. This might help to improve the design of randomized controlled trials to test alternative interventions for patients with high risk of non-RTW. However, our model also needs external validations studies in different settings (case-mix, insurance environment etc.) and impact studies on clinical practice [63]. On the other hand, the discriminative ability could probably be improved by introducing simple questions on patients’ jobs expectations [23], [72], [81].

### Conclusion

This validated prediction model allows the estimation of the probability of non-return to work for patients requiring occupational rehabilitation after orthopaedic trauma. This model, the Wallis Occupational Rehabilitation RisK (WORRK) model, presents only 19 items easily assessed in a clinical setting. It has moderate discriminative ability, adequate calibration, is useful for all kinds of trauma and is applicable to vulnerable populations like immigrant workers. This makes this model informative for physicians and multidisciplinary teams managing such patients and may facilitate research in this domain by enabling the study of patients with similar risk profiles.

## Supporting Information

Reduced Model S1 **The WORRK Model and Probability Risk Score.**
(PDF)
