# Correction: Predicting Non Return to Work after Orthopaedic Trauma: The Wallis Occupational Rehabilitation RisK (WORRK) Model

**DOI:** 10.1371/journal.pone.0119193

**Published:** 2015-03-05

**Authors:** The PLOS ONE Staff

There is an error in the layout of question 7 in S1 Reduced Model. Please see the corrected file, renamed S1 Reduced Model, below.

## Supporting Information

S1 Reduced Model The WORRK Model and Probability Risk Score. (PDF)
