# Supplementary material for: Predicting Non Return to Work after Orthopaedic Trauma: The Wallis Occupational Rehabilitation RisK (WORRK) Model
Source: PLoS One. 2014 Apr 9;9(4):e94268. doi: 10.1371/journal.pone.0094268 (PMC3981787; doi:10.1371/journal.pone.0094268)
Supplement: Reduced Model S1 — The WORRK Model and Probability Risk Score. (PDF) [file pone.0094268.s001.pdf]

Reduced Model S1: The Wallis Occupational Rehabilitation RisK (WORRK) model and Probability Risk Score

**Patient's personal data**		
1. Age : ☐☐ yrs	2. Education ≥ 9 yrs : ☐ Y / ☐ N	3. Professional certification: ☐ Y / ☐ N
4. Work related injury : ☐ Y / ☐ N	5. Local native language: ☐ Y / ☐ N	

**Patient's perception data**		
6. Pain	no pain ————————————————	the worst pain
7. Quality of Life	the best it has ever been ————————————————	the worst it has ever been

**Patient's biopsychosocial data**				
	☐ 0	☐ 1	☐ 2	☐ 3
8. Chronicity	Less than 3 months of physical dysfunctionning	More than 3 months of physical dysfunctionning or several period of less than 3 months	A chronic disease	Several chronic disease
	☐ 0	☐ 1	☐ 2	☐ 3
9. Restrictions in coping	No restrictions in coping; ability to manage stress adequately, no impairment of medical treatment	Mild restrictions in coping which causes mild to moderate distress in patients and/or relatives or health care providers (such as complaining behaviour)	Moderate restrictions in coping which causes severe emotional distress in patients and/or relatives or health care providers and/or impairment of medical treatment	Severe limitations in coping which produces serious psychiatric symptomatology such as substance abuse, self-mutilation or attempted suicide and impairment of medical treatment
	☐ 0	☐ 1	☐ 2	☐ 3
10. Resistance to treatment	Interested in receiving treatment and willing to cooperate with treatment	Some ambivalence though willing to cooperate to treatment	Considerable resistance, such as non-compliance, hostility or indifference toward health care professionals	Active resistance against medical care
	☐ 0	☐ 1	☐ 2	☐ 3
11. Psychiatric symptoms	No psychiatric symptoms	Mild psychiatric symptoms such as problem to concentrate or feeling tense	Psychiatric symptoms such as anxiety, depression or confusion	Psychiatric symptoms with behavioural disturbances, such as violence or self-inflicting behaviour
	☐ 0	☐ 1	☐ 2	☐ 3
12. Restrictions in integration	A job (including housekeeping, retirement, studying) and having leisure activities	A job (including housekeeping, retirement, studying) without leisure activities	Unemployed now and for at least 6 month with leisure activities	Unemployed now and for at least 6 month without leisure activities
	☐ 0	☐ 1	☐ 2	☐ 3
13. Social dysfunctionning	No social disruption	Mild social dysfunction, interpersonal problems	Moderate social dysfunction, such as inability to initiate or maintain social relations	Severe social dysfunction, such as involvement in disruptive social relations or social isolation
	☐ 0	☐ 1	☐ 2	☐ 3
14. Restriction of network	Good contacts with family, work and friends	Restriction in one of the domains	Restrictions in two of the domains	Restrictions in three of the domains

	☐ 0	☐ 1	☐ 2	☐ 3
15. Organisation of care	Primary care/general practitioner only	Different specialists from the general health care system	Both general health care and mental health care service	Hospitalization or transfer from a hospital
	☐ 0	☐ 1	☐ 2	☐ 3
16. Complications & Life Threat	No risk of limitations in activities of daily living	Mild risk of limitations in activities of daily living	Moderate risk of permanent limitations of activities in daily living	Severe risk of physical complications with serious permanent functional deficits and/or dying
	☐ 0	☐ 1	☐ 2	☐ 3
17. Mental health threat	No risk of psychiatric disorder	Mild risk of psychiatric symptoms, such as stress, anxiety, feeling blue, substance abuse or cognitive disorder; mild risk of treatment resistance (ambivalence)	Moderate risk of psychiatric disorder requiring psychiatric care; moderate risk of treatment resistance	Severe risk of psychiatric disorder requiring frequent visits and/or hospital admissions; risk of refusal treatment for serious psychiatric disorder
	☐ 0	☐ 1	☐ 2	☐ 3
18. Social Vulnerability	No risk of changes in the living situation; adequate social support and integration	No risk of changes in the living situation but additional social support and/or job accommodation	Risk of changes in the living situation, such as temporary admission to facility/institution and/or vocational guidance	Risk of need for permanent admission to facility/institution and/or permanent disability pension
	☐ 0	☐ 1	☐ 2	☐ 3
19. Coordination of health care	No problems in the organization of care	Mild efforts needed to organize care: multidisciplinary care which is quite easy to organize, e.g. distant service access	Moderate efforts to organize care: multidisciplinary care which is difficult to organize, e.g. potential insurance loss, communication barriers	Severe efforts needed to organize care, e.g. little or no insurance, resistance to communication and coordination among providers

The probability not returning to work can be calculated with the formula:

Probability Risk Score: = 1/[1 + exp(- scoring function)]

where the scoring function equals to -2.649848 + 0.0179208 * Age, per one year + Education > 9 yrs * -0.2112931 + Work related injury * 0.1079563 + Local native language * -0.0857251 + Qualified work * -0.4087628 + Pain * 0.0069819 + Quality of Life * -0.006264 + Chronicity * -0.0287237 + Restrictions in coping * 0.0522402 + Resistance to treatment * 0.1753309 + Psychiatric symptoms * -0.0406474 + Restrictions in integration * 0.3793558 + Social dysfunctionning * 0.0095086 + Restriction of network * 0.3085014 + Organisation of care * 0.2552069 + Complications & Life Threat * 0.4628969 + Mental health threat * -0.0729892 + Social Vulnerability * 0.0729917 + Coordination of health care * 0.1123762

The coefficients are based on both samples with 1423 (development sample) + 819 (validation sample) patients.
